# Antinociceptive properties of losmapimod in two acute pain models in rats: behavioural analysis, immunohistochemistry, dose response, and comparison with usual analgesic drugs^☆^

**DOI:** 10.1016/j.bjao.2022.100029

**Published:** 2022-08-17

**Authors:** Mickaël Soued, Leila Hamdi, Mouna Ben Rehouma, Jean-Xavier Mazoit, Dan Benhamou

**Affiliations:** 1 Laboratory of Anaesthesia, Inserm U 1195 Neuroprotection et neurorégéneration, Paris-Saclay University, Le Kremlin-Bicêtre, France; 2 Department of Anaesthesia, Antoine Béclère Hospital, APHP, Paris-Saclay University, Clamart, France; 3 Department of Anaesthesia and Intensive Care Medicine, Bichat Hospital, APHP, Paris Seine Saint Denis, Paris, France; 4 Department of Anaesthesia and Intensive Care Medicine, Bicêtre Hospital, APHP, Paris-Saclay University, Le Kremlin-Bicêtre, France

**Keywords:** acute pain, antinociceptive drug, losmapimod, p38 inhibition, rodent

## Abstract

**Background:**

The p38 protein is a ubiquitous mitogen-activated protein kinase involved in the proinflammatory signalling pathway and in the pain response after various noxious stimuli. Many p38 inhibitors have been developed and shown to provide effective analgesia in animal models. They are, however, mainly administered intrathecally or intravenously. Our study aimed to evaluate losmapimod, a novel oral p38 inhibitor, in two murine acute pain models.

**Methods:**

Losmapimod (12 mg kg^−1^) was compared with paracetamol, ketamine, and morphine using thermal and mechanical stimulation after carrageenan injection. A dose–effect study was also performed with this model. Behavioural testing was also performed in a plantar incision model to confirm the analgesic effect of losmapimod. Expression of activated p38 in neurones, microglia, and astrocytes was also investigated at 2, 15, and 24 h after carrageenan injection.

**Results:**

Losmapimod was both antiallodynic and antihyperalgesic in the carrageenan pain model and provided an antinociceptive effect similar to that of morphine. The dose of 12 mg kg^−1^ was shown to be the ED_78_ and ED_64_ after thermal and mechanical stimulation, respectively. After plantar incision, losmapimod provided a significant antinociceptive effect. No life-threatening side-effect was observed in the behavioural study. Losmapimod prevented neurone and microglial activation at 2 and 15 h after carrageenan injection, respectively, but no effect was found on astrocytic activation.

**Conclusion:**

Losmapimod appears to be a promising drug in severe acute pain conditions. Losmapimod could also be helpful for postoperative pain control, as suggested by its effect after plantar incision.

The mitogen-activated protein kinase (MAPK) system is a complex system with widespread distribution in the body, involved in the control of many key cell pathways,^1^^,^^2^ and especially in proinflammatory cytokine synthesis pathways.^3^, ^4^, ^5^ Among many different cell types, microglial and neuronal cells express the cellular p38 MAPK pathway. After many different peripheral stimuli such as nerve injury or peripheral inflammation, the neuronal level of phosphorylated p38 (p-p38)/p38 increases,^6^, ^7^, ^8^, ^9^ leading to neuronal secretion of various chemokines which contribute to microglial activation.^9^^,^^10^ The subsequent perineural inflammation activates all other glial cells^9^, ^10^, ^11^ and produces allodynia or hyperalgesia.^6^, ^7^, ^8^^,^^12^, ^13^, ^14^ p38 MAPK thus plays a crucial role in pain response and spinal cord sensitisation.^11^^,^^15^ Consequently, p38 MAPK might be a therapeutic target for pain treatment. Several MAPK inhibitors (MAPKi) have been developed and have demonstrated efficacy by alleviating allodynia and hyperalgesia in animals^6^^,^^14^, ^15^, ^16^, ^17^ and postoperative dental pain in humans.^18^ Some studies have, however, provided contradictory results in chronic pain patients,^19^^,^^20^ suggesting that acute pain might be a better target. A minority of MAPKi can be administered orally, and those inhibiting the p38 α subunit have provided mixed results regarding pain relief and dose-related toxicity in human studies.^21^ Conversely, losmapimod inhibits both p38 α and β subunits and was very well tolerated in animals and humans.^22^ As among currently available antinociceptive drugs, opioids are associated with many side-effects, some of which are life-threatening, it seems appropriate to search for drugs that may replace them or have an opioid-sparing effect.

This study included several parts. The first part was based on a carrageenan-induced acute pain model. Carrageenan injection in the rat paw is a well-known model of enhanced microglial phosphorylation of p38^17^ by provoking acute peripheral pain^23^ with its thermal and mechanical components.^12^^,^^24^ We initially assessed the overall effect on pain with behavioural tests and compared its efficacy with that of morphine and other analgesic and antihyperalgesic drugs. Because only one effective dose of losmapimod has been used in a murine model,^25^ we then determined the dose–response curve of losmapimod. Immunohistochemistry was used to evaluate losmapimod-induced inhibition of p38 activation in the dorsal root ganglia (DRG) and the spinal cord dorsal horn. Because doubts have been expressed as to the value of murine pain models to predict efficacy in humans,^26^ we assessed the effects of losmapimod in the plantar incision model, another well-defined but mechanistically different pain model.

## Methods

All experiments were approved by our Ethical Animal Committee (CEEA 26, Paris-Sud, No. 4889/2016041110429969 and 15570/2018101210036429, accepted on 6 June 2016 and 15 December 2018, respectively) and conducted according to the Animal Research: Reporting of *In Vivo* Experiments (ARRIVE) guidelines. Male Sprague–Dawley rats (Janvier Labs, Le Genest-Saint-Isle, France) weighing 280–300 g were housed by groups of three at 22°C (2°C) with food and water available *ad libitum* and maintained on a 12-h light–dark cycle. All procedures were performed under isoflurane anaesthesia. After testing, all animals were euthanised using an overdose of pentobarbital sodium.

### Tests used in behavioural approach

After 1 week of habituation and training, rats were subjected to behavioural testing. Nociception to mechanical stimulation was measured using von Frey hairs. Starting from the softer hair, application was performed 10 times by filament size with incremental stiffness if the rat did not withdraw its paw. The test was considered positive when withdrawal occurred at least six times per 10 applications. Nociception to heat was measured using the Hargreaves plantar test. A radiant heat source was focused under the injury site with a 20-s cut-off to avoid tissue damage. The mean value of three consecutive trials was used. After acclimatisation, all different test measurements were performed once daily from Day 0 (D0) to Day 5 (D5) inclusive.

Side-effects were defined as modification of rat behaviour. Serious side-effects were defined as emergence of signs of distress in accordance with the usual rules of animal well-being (mutilation, weight loss up to 10%, among others), or death.

### Immunohistochemistry

Rats were overdosed with sodium pentobarbital (intraperitoneal) and intracardially perfused with 0.9% NaCl, followed by 4% paraformaldehyde. Tissues were paraffin-embedded and sectioned (7 μm thickness). Sections were incubated with a rabbit polyclonal anti-p38 phosphorylated antibody (ab4822, 1:250; Abcam, Cambridge, UK) and a mouse monoclonal Anti-GFAP antibody (AMAB91033, 1:500; Sigma, St. Louis, MO, USA), anti-NeuN antibody (ab104224, 1:1000; Abcam), or Anti-CD11b/c antibody [OX42] (ab1211, 1:100; Abcam). Sections were then rinsed and incubated in a secondary antibody solution (A-21428, 1:1000, or A-11029, 1:1000; Life Technologies, Saint-Aubin, France), washed and were counterstained with 4,6-diamidino-2-phenylindol dihydrochloride (DAPI) 0.5 μg ml^−1^ (Sigma, St-Quentin-Fallavier, France). The DAPI primary antibody-stained slices in blue after immunofluorescence, whereas phosphorylated p38 induced red and cell markers (GFAP, NeuN, and CD11b) induced green staining. Activated cells were thus triple stained. Images were acquired at 20× magnification. ImageJ freeware (1.8.0_172; National Institutes of Health and the Laboratory for Optical and Computational Instrumentation, USA, https://imagej.nih.gov/ij/download.html) was used to read immunofluorescent acquisitions and count positive cells on each slide.

Slides were analysed by two different investigators (blinded to each other) and the mean value was retained for statistical analysis. Because the total number of neurones in DRG varies between rats and between sides, the percentage of neurones expressing p-p38 was thus retained for analysis. The percentage of activated neurones was defined as total number of activated neurones divided by the total number of left DRG neurones. The percentage of microglial and astrocytic cells was not obtained (the count of inactivated microglial and astrocytic cells is technically impossible); we thus compared the number of activated cells ipsilateral to the injury with the number contralaterally, with the assumption that rats have a symmetrical spinal cord.

### Experimental protocols

Carrageenan 3% (Sigma, Saint-Quentin-Fallavier, France) diluted in saline (100 μL, NaCl 0.9%) was administered subcutaneously once, on D0, in the left hind paw. The following drugs were administered: losmapimod (Euromedex, Strasbourg, France; 12 mg kg^−1^ orally^25^), paracetamol (acetaminophen) (Panpharma, La-Selle-en-Luitré, France; 500 mg kg^−1^ orally^27^), ketamine, (Panpharma; 20 mg kg^−1^, s.c.^28^), and morphine (CDM Lavoisier, Paris, France; 3 mg kg^−1^ s.c.^29^). S.C. drug injection was performed in the abdominal wall using a 16 mm length, 25G diameter needle. A feeding canula introduced in the oesophagus was used for oral administration and considered successful if the rat did not spit or cough. If administration failed, the rat was excluded from further analysis.

Six groups were studied: carrageenan alone, losmapimod alone, and one of four combinations: carrageenan+losm-apimod, carrageenan+morphine, carrageenan+paracetamol, carrageenan+ketamine. All analgesic drugs were administered once daily, 60 min before experiments on D0, D1, and D2.

A losmapimod dose–response curve was modelled for both mechanical and thermal stimulation. Considering that the previously described dose was in the range of efficacy,^25^ five groups were studied with the following incremental doses: 0, 2, 4, 12, or 50 mg kg^−1^, orally, once a day from D0 to D2.

Lastly, plantar incision was performed, as a postoperative pain model on D0, as previously described by Brennan and colleagues.^30^ Losmapimod was administered once a day, orally as described above, from D0 to D2 using the ED_90_ from the previously obtained dose response study. von Frey hair application and the Hargreaves test were performed after carrageenan injection, that is once a day, from D0 to D5. In this part, two groups were studied, comparing plantar incision with and without losmapimod.

Immunochemistry measurements included two separate groups (carrageenan either alone or associated with losmapimod). The percentage of neurones expressing p-p38 was measured in the fourth lumbar (L4) left DRG at 2 h and 15 h. The number of microglial cells and astrocytes expressing p-p38 was measured in the L4–L5 dorsal horn of the spinal cord at 15 h and 24 h.

### Statistical analysis

The dose–effect of losmapimod (*E*) was fitted using a simple *E*_max_ model (*E*=*E*_0_+*E*_max_×*D*/(ED_50_+*D*), where *E*_0_ and *E*_max_ are the basal and maximum theoretical effects, respectively; *D* is the dose administered; and ED_50_ is the dose leading to half-maximum effect (*E*_max_–*E*_0_)/2). We used NONMEM® version VI (NONlinear Mixed Effects Model; ICON Clinical Research LLC, Dublin, Ireland) with a simple multiplicative error parameter. The estimated 95% confidence interval (CI) was calculated using log-likelihood profiling. Data are described to three significant digits.

The minimum number of animals in each group was previously calculated considering an 80% power and a 5% α risk (bilateral, corrected for the number of planned comparisons using the Bonferroni correction). The effect size corresponding to each experiment was calculated using previous data obtained in our laboratory.

All statistical analyses were performed using the R software (R Core Team 2018, Vienna, Austria; https://cran.r-project.org/). Normality of data was tested using qq plots and the Shapiro–Wilk test. Because behavioural data were not normally distributed, we used the package nparLD which is an analysis of variance (anova) on ranks which uses the marginal distribution of ranks, with time as repeated measures and drugs as factor. Between groups comparisons were corrected using the Bonferroni–Holm method. To avoid any error inflation, we did not compare the groups at specific times. Behavioural baseline data (D0) were compared using the Kruskal–Wallis test.

All other data (percentage of activated neurones in DRG and number of microglial and astrocytic activated cells) were normally distributed with equal variances and were compared using anovas (two-way for DRG and three-way for spinal cord with side as a within-group factor and treatment and time as between-group factors).

Data are expressed as median [inter-quartile range] or mean (standard deviation [sd]), as appropriate. *P*<0.05 was considered the minimum level of significance.

## Results

### Behavioural study and evaluation of losmapimod antinociceptive properties

#### Antinociceptive properties after carrageenan injection

**Fig 1:**
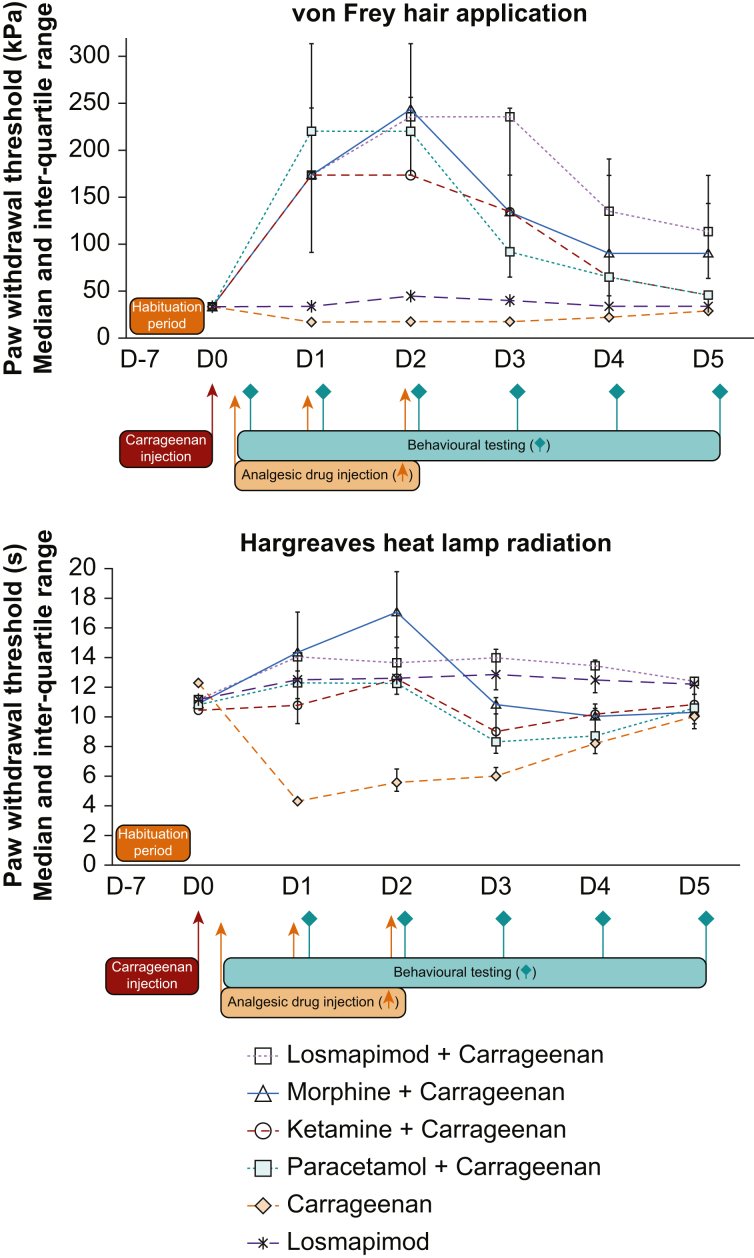
Behavioural results after carrageenan injection. Nine rats were included in each group. Paw withdrawal threshold after von Frey hair stimulation (upper panel) and Hargreaves heat lamp application (lower panel) as a function of time. Data are expressed as median and inter-quartile range. To improve the clarity of the figure after von Frey hair application, values in ‘losmapimod+carrageenan’ and ‘paracetamol+carrageenan’ groups were slightly modified (from 245 to 235 kPa on D2 and D3, and from 245 to 220 kPa on D1 and D2, respectively). Briefly, carrageenan 3% 100 μl was administered subcutaneously in the left hind paw. Ketamine 20 mg kg^−1^ and morphine 3 mg kg^−1^ were also administered subcutaneously. Paracetamol 500 mg kg^−1^ and losmapimod 12 mg kg^−1^ were administered orally. *P*<0.01 for comparison between ‘losmapimod+carrageenan’ group and all other groups after Hargreaves test. After von Frey hair stimulation; *P*<0.01 for comparison between the ‘losmapimod+carrageenan’ group and all others except the ‘morphine+carrageenan’ group where the difference did not reach significance.

Nine rats were included in each group. After habituation, baseline values were stable for at least 2 days before D0 (Fig. 1). We were unable to demonstrate any difference between groups at baseline for mechanical allodynia and thermal hyperalgesia.

Carrageenan induced significant mechanical allodynia and thermal hyperalgesia (*P*<0.001). When administered alone, losmapimod significantly increased paw withdrawal threshold after mechanical and thermal stimulation (*P*=0.003 and *P*=0.002, respectively). Losmapimod, morphine, paracetamol, and ketamine significantly reversed the effect of carrageenan (*P*<0.0001 for all drugs and for the two tests).

Morphine allowed a similar reduction of mechanical hyperalgesia to losmapimod (*P*=0.26). Paracetamol and ketamine both reduced the pronociceptive effect of carrageenan but with a less marked effect than losmapimod.

### Dose–response curves after carrageenan injection

**Fig 2:**
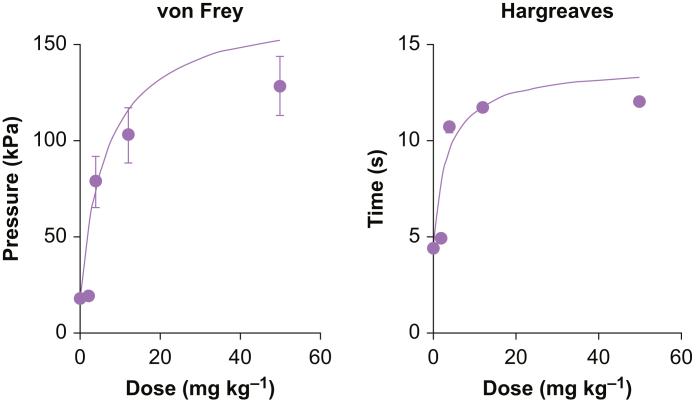
Dose–response curves of losmapimod-induced antinociceptive effect using the Hargreaves (left panel) and von Frey hair (right panel) tests. C represents the losmapimod concentration. Each point represents one dose (0, 2, 4, 12, and 50 mg kg^−1^).

Thirty-two rats were included (eight rats in the 0 mg kg^−1^ group and six rats in all other groups) (Fig. 2). Data obtained on D1 were used because the greatest effect of losmapimod was observed at this time point in all groups. For mechanical allodynia, *E*_0_, *E*_max_, and ED_50_ with their 95% CI were 19.3 kPa, 151.0 kPa, and 6.8 (2.1–19.6), mg kg^−1^, respectively. For thermal hyperalgesia, the corresponding values were 4.4 s, 13.9 s, and 3.4 (1.4–6.9) mg kg^−1^. The dose of 12 mg kg^−1^ was therefore the ED_64_ and ED_78_ for mechanical allodynia and thermal hyperalgesia blocking effect, respectively.

### Plantar incision

**Fig 3:**
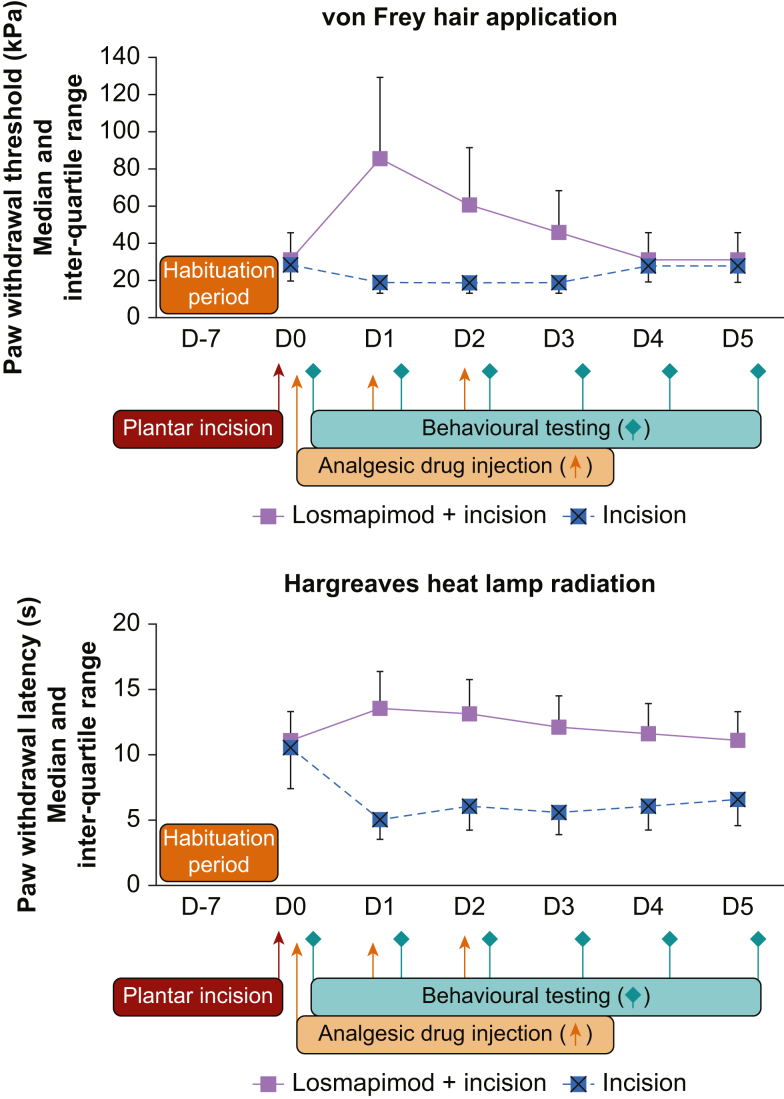
Behavioural results after plantar incision. Paw withdrawal threshold after von Frey hair stimulation (upper panel) and Hargreaves heat lamp application (lower panel) as a function of time. Data are expressed as median and inter-quartile range. Losmapimod was administered orally at 30 mg kg^−1^. The difference was statistically significant (*P*<0.01) between the two groups, both after mechanical and thermal stimulation.

After plantar incision, losmapimod was administered at 30 mg kg^−1^, approximately corresponding to the antinociceptive ED_90_ (ED_89_ and ED_82_ for Hargreaves and von Frey tests, respectively) (Fig. 3). Nine rats were included in each group (plantar incision alone and plantar incision associated with losmapimod). Allodynia and hyperalgesia induced by plantar incision (*P*<0.01 for both tests) were reversed by losmapimod administration, which enhanced paw withdrawal thresholds (*P*<0.01 for both tests).

### Side-effects

No serious side-effect was noted during the whole study. For rats included in the ketamine+carrageenan group, behavioural testing had to be delayed because of altered and uncooperative behaviour, leading to difficulties in obtaining reliable paw withdrawal thresholds. Tests were therefore performed 90 min after ketamine administration (instead of 60 min in other groups). During the dose–response study, one rat included in the 50 mg kg^−1^ group had alternating constipation and diarrhoea. Sedation was not observed in any animal receiving losmapimod. No other side-effect was recorded.

### Immunohistochemistry

**Fig 4:**
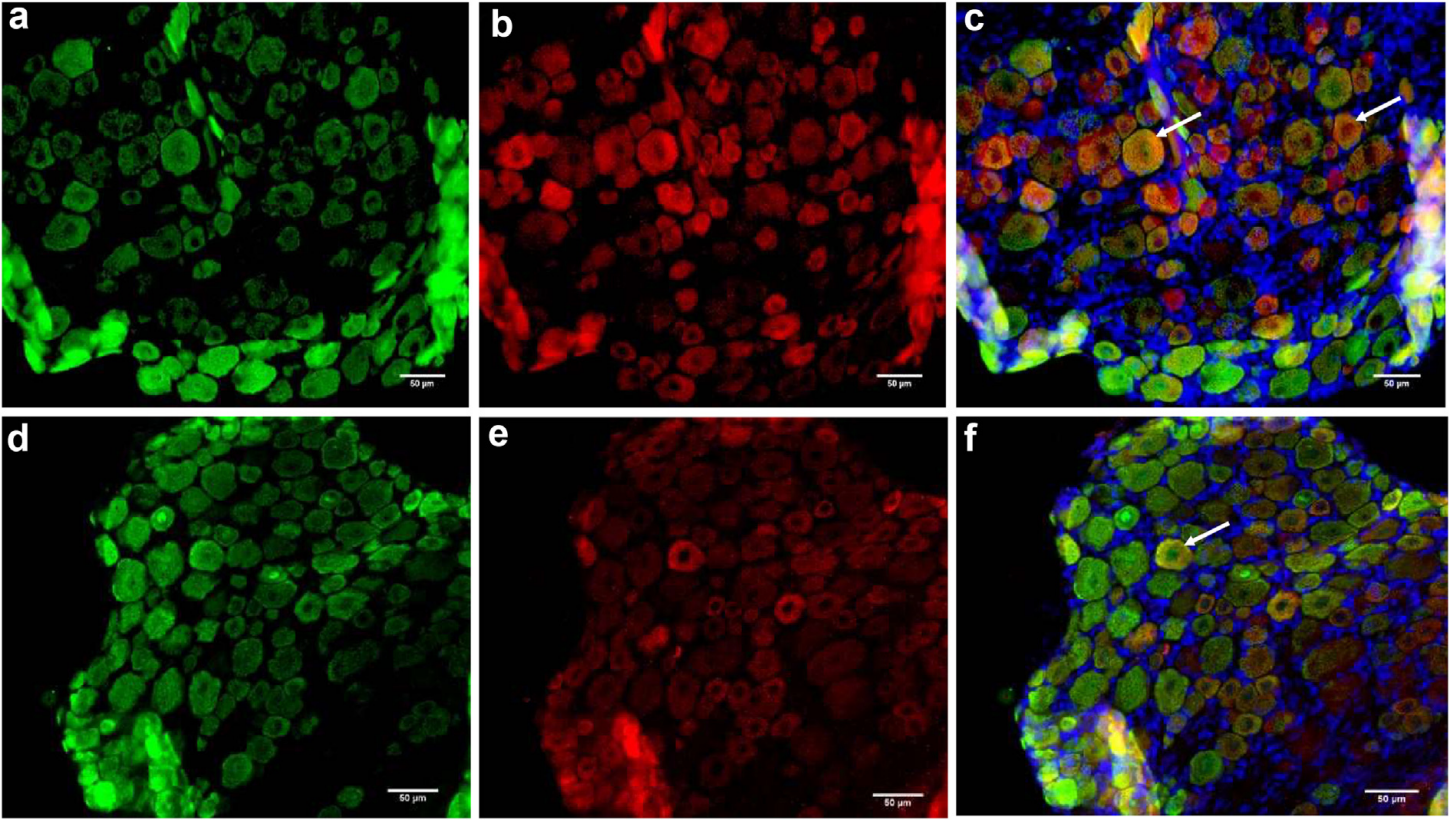
Phosphorylated p38 (p-p38) expression in left DRG neurones at 2 h in ‘carrageenan’ (a to c) and ‘losmapimod+carrageenan’ group (d to f). Neurones are stained in green (Neu-N marker) (a and d), phosphorylated p38 is stained in red (b and e), and blue corresponds to DAPI staining (unspecific marker of cell nucleus). Triple-stained cells were counted and appear in yellow (c and f). Examples of triple-stained cells are pointed with white arrows (c and f). The white scale bar corresponds to 50 μm. DAPI, 4,6-diamidino-2-phenylindol dihydrochloride.

**Fig 5:**
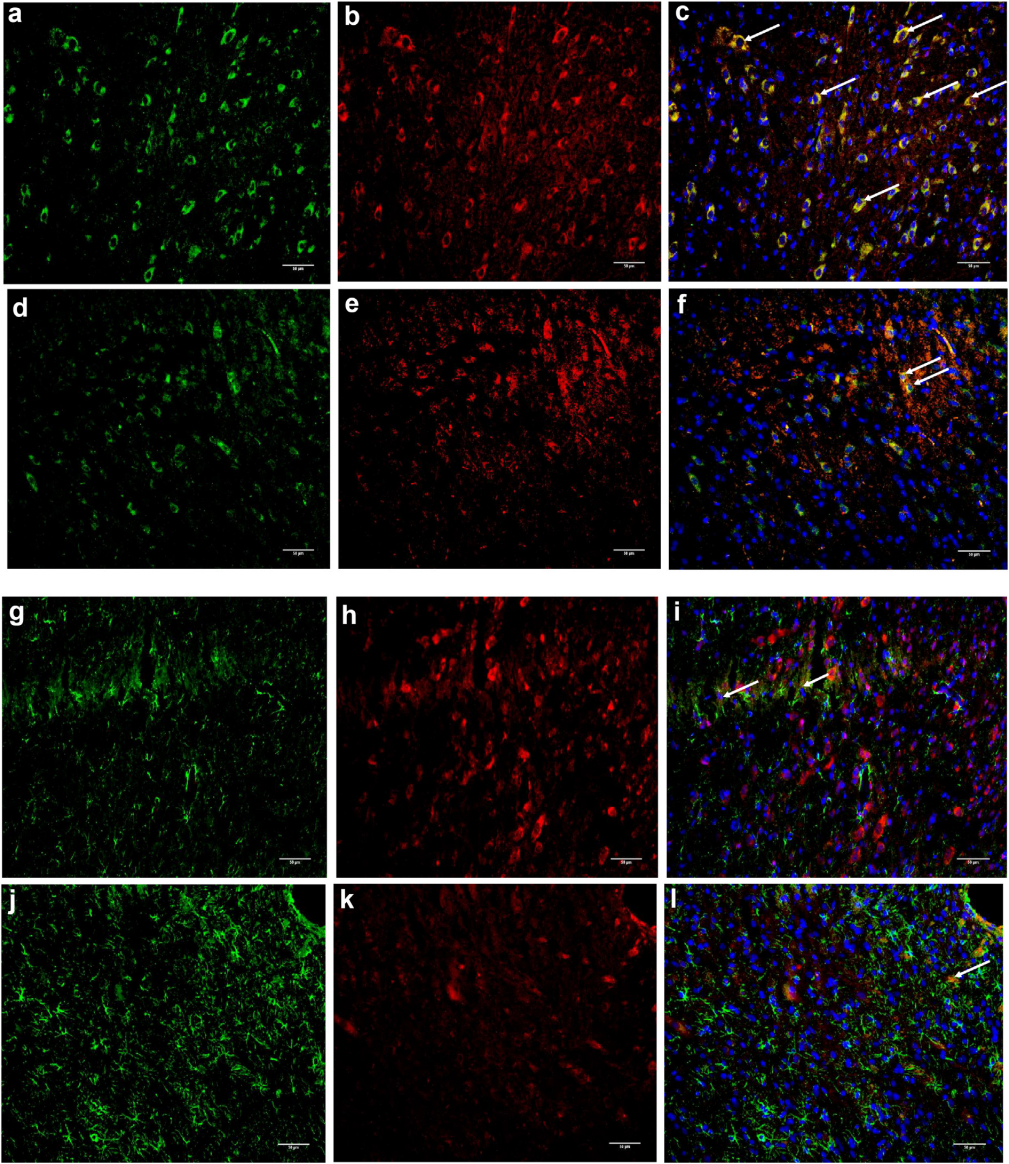
Ipsilateral (left side of spinal cord) phosphorylated p38 expression in microglia (a to f) and astrocytes (g to l) at 15 h in ‘carrageenan’ (a to c and g to i) and ‘losmapimod+carrageenan’ (d to f and j to l) groups. Microglial and astrocytic cells are stained in green (CD11b and GFAP, respectively) (a, d, j, and l), phosphorylated p38 is stained in red (b, e, h, and k) and blue corresponds to DAPI staining (unspecific marker of cell nucleus). Triple-stained cells were counted and appear in yellow (c, f, i, and l). Examples of triple-stained cells are pointed with white arrows (c, f, i, and l). The white scale bar corresponds to 50 μm. DAPI, 4,6-diamidino-2-phenylindol dihydrochloride; GFAP, glial fibrillary acidic protein.

Two hours after injury, losmapimod significantly decreased the percentage of activated neurones in the DRG (*P*=0.0004) (Table 1 and Fig 4, Fig 5). However, this effect was transient (*P*=0.0003).

**Table 1:** Number of cells expressing phosphorylated p38 (p-p38) in neurones in DRG, microglia or astrocytes in spinal cord, 2, 15, and 24 h after carrageenan insult in the left paw. Because the total amount of neurones in each DRG varies between rats and between sides, the percentage of neurones expressing p-p38 was retained for analysis. Carr, carrageenan; Carr+Losma, carrageenan+losmapimod; *n*, number of rats in groups; sd, standard deviation.

	2 h		15 h		24 h	
	Carr	Carr+Losma	Carr	Carr+Losma	Carr	Carr+Losma
**Dorsal root ganglion (activated cells, percentage per field [**sd**])**						

Neurones	90 [10] (*n*=5)	50 [18] (*n*=7)	91 [10] (*n*=6)	91 [10] (*n*=6)		
**Spinal cord (number of activated cells per field [**sd**])**						

Microglia Ipsilaterally (left) Contralaterally (right)			43 [18] 19 [11]	20 [9] 14 [9]	8 [9] 6 [7]	7 [6] 5 [5]
Astrocytes Ipsilaterally (left) Contralaterally (right)			30 [17] 20 [11]	29 [15] 27 [13]	11 [7] 5 [4]	8 [4] 5 [3]

In the dorsal horn of the spinal cord, carrageenan induced a significant microglial activation (*P*<0.0001, ipsi-*vs* contralaterally), which was suppressed by losmapimod (*P*=0.030). However, both the activation induced by carrageenan and the suppressive effect of losmapimod were transient and no difference in any group was observed 24 h after injury (*P*<0.0001). In addition, all interactions were significant (*P*<0.0001, *P*=0.001, and *P*=0.003 for the side×time, side×group, and side×time×group interactions, respectively).

Carrageenan also induced significant astrocyte activation (*P*<0.0001; ipsi-*vs* contralaterally). This activation significantly decreased with time (*P*<0.0001 24 *vs* 15 h after insult), but losmapimod had no effect (*P*=0.85).

## Discussion

In this study performed in murine acute pain models, we showed that: (1) losmapimod demonstrated potent inhibition of the pain behaviour induced by carrageenan similar to that produced by morphine but stronger than ketamine and paracetamol; (2) losmapimod reduced neuronal and microglial activation 2 and 15 h, respectively, after carrageenan administration; (3) large doses were necessary to reach full antinociceptive activity; (4) these results were strengthened by the plantar incision approach, which provided similar behavioural results.

In the carrageenan pain model, losmapimod provided an effective antinociceptive effect in all different behavioural tests. The potency of pain relief obtained with other antinociceptive drugs tested is in accordance with previous studies.^27^^,^^29^ However, despite a similar duration of drug administration, losmapimod provided a more persistent antinociceptive effect, with a less marked down-sloping effect of paw withdrawal after the last drug administration. Conversely, in the morphine group, after an initially potent antinociceptive effect, the paw withdrawal threshold decreased sharply after D2, particularly after thermal stimulation. This could be related to the occurrence of opioid-induced hyperalgesia, even after a very small number of opioid doses.^31^^,^^32^

Ketamine fully reversed the pronociceptive effect of carrageenan after thermal stimulation and demonstrated an antinociceptive effect using the von Frey test. However, in both cases, losmapimod appeared more potent and had a longer duration of action. Ketamine also caused behavioural changes which delayed our experiments.

Losmapimod administration led to an increase in paw withdrawal threshold above baseline after both thermal and mechanical tests, conversely to results obtained with other p38 inhibitors (FR167653, SD-282, or SB203580),^6^^,^^7^^,^^15^^,^^33^ which partially^6^^,^^33^ or fully^7^^,^^15^ reversed the antinociceptive effect of the noxious stimulus. However, as inhibition of thermal and mechanical stimuli is dose-related,^6^^,^^7^ doses might not be equipotent and the p38 subunit targeted could vary (α, β, or both).

The potent antinociceptive effect seems at odds with human studies where losmapimod failed to reverse pain. Apart from the often-mentioned species difference which might explain the discrepancy, several factors might be at play. First, two negative human studies^19^^,^^20^ explored patients who were in chronic pain, and neurophysiological mechanisms might well be different. As noted by Ostenfeld and colleagues^20^ themselves, MAPK activation occurs early with microglial activation in the DRG and spinal cord and contributes to the development of neuropathic chronic pain. Once these long-standing mechanisms have been established, it is possible that pharmacological MAPK inhibition becomes less effective and cannot reverse this chronic inflammatory state. Our immunohistochemistry results also suggest that losmapimod demonstrated an early inhibition of the afferent pathway in the DRG and spinal cord by reducing neurone firing very early (at 2 h) and microglia activation at 15 h. In the only human study performed in patients with acute dental pain, an orally active p38 α MAPKi was effective.^18^ Another plausible explanation for the ineffectiveness of losmapimod in these previous studies could be related to the dose used in the human chronic lumbosacral radiculopathy study,^20^ which might also have been too low to counteract the inflammatory neuropathic pain. The authors used a dose (i.e. 7.5 mg twice daily) with the aim of obtaining a dose of losmapimod equivalent to 0.1–10 mg kg^−1^ in rats. Our dose–response study demonstrated that the ED_90_ is closer to 30 mg kg^−1^. Finally, antinociceptive properties of losmapimod found after carrageenan injection were confirmed after plantar incision, providing further evidence that MAPKis are effective in acute pain.

Losmapimod displayed a dose-related effect which was more potent on thermal allodynia than on mechanical hyperalgesia. This discrepant effect could be linked to the production of prostaglandin E_2_, which produces more thermal hyperalgesia compared with mechanical allodynia at similar levels.^34^ Similar to losmapimod, other p38 inhibitors (FR167653, SD-282, and SB203580) also have dose-related antinociceptive effects, but complete dose–response curves had not yet been constructed.^7^^,^^15^^,^^33^ Our dose response study, strengthened by few previous animal and human studies,^19^^,^^20^^,^^22^^,^^25^ suggests that losmapimod dosing could be safely increased, in contrast to the other drugs we tested.

Most studies have highlighted that neurone activation leads to microglial sensitisation after peripheral noxious stimuli and suggest that astrocytes are activated secondarily.^35^ We chose three different time points (2, 15, and 24 h after carrageenan injection) with the hope to best fit with progressive activation of neurones, followed by microglia and astrocytes.^35^

Carrageenan significantly enhanced expression of phosphorylated p38 at 2 and 15 h, but losmapimod only prevented this activation at 2 h in neurones. In some human studies, losmapimod has been administered twice daily^19^^,^^20^ and an elimination half-life of around 9 h has been described.^22^ The elimination half-life remains unknown in rats. In the present study, losmapimod was initially administered using a dosing scheme similar to that previously used (12 mg kg^−1^ once a day).^25^ The lack of p38 inhibition in neurones at 15 h might also be explained using a small dose compared with the ED_90_ of 30 mg kg^−1^ we found in our dose–response study.

A significant microglial activation was observed, which was reduced after losmapimod administration at 15 h but not at 24 h. As microglial activation had already decreased by 24 h, any effect of losmapimod was hard to discern.

Astrocyte activation observed at 15 h was less marked at 24 h. Contrary to our expectations, losmapimod did not modify the time course of astrocyte activation. A weaker enhancement of p38 phosphorylation in astrocytes compared with microglial cells has been previously described^8^^,^^14^^,^^15^ The peak of astrocyte activation or the intensity of losmapimod-induced inhibition possibly occurred before 15 h, and at this time cell activation could be on a decreasing trend. Measurement of other markers, downstream to p38 in the inflammatory pathway, in the spinal cord or in peripheral areas might have been more appropriate. Further studies could thus be helpful.

The dose–response study has several limitations. First, the side-effect rate could have been underestimated, mainly because subjective testing of rats is not easy. Also, because of the small number of data points (four doses), we did not model the concurrent effect of carrageenan and losmapimod using an inhibitory *E*_max_ model.^36^

Assessment of pain function using behavioural testing in rodents has also been criticised because the all-or-nothing response lacks precision.^37^ In addition, numerous drugs have been shown to be efficient in animals but not in humans. We indeed agree that our results should be interpreted with caution,^26^ but they represent the first step that is needed to describe the antinociceptive efficacy of a new class of drugs. In addition, immunochemistry measurements, the dose–response study and comparison with other well-known antinociceptive drugs were used to better investigate the efficacy of losmapimod. The comparison between losmapimod and other drugs might not have used equipotent doses. However, because data on the most effective dose were lacking for losmapimod, we could only compare the only previously published dose of losmapimod in rats to the highest effective doses devoid of side-effects of paracetamol,^27^ ketamine,^28^ and morphine.^29^ Lastly, our results were confirmed in a second acute pain model, closer to daily clinical practice. Plantar incision is well validated^30^ as a postoperative pain model. In this part of the study, a group with losmapimod alone (without plantar incision) was not included, and we recognise that had we used 30 instead of 12 mg kg^−1^, different results could have been obtained. Plantar incision was, however, performed as a validation sub-study, and using an additional dose would have required more animals to be studied. However, losmapimod provided similar antinociceptive effects in both pain models.

A sham group was not included. However, all experiments were started after obtaining a stable paw withdrawal threshold. Moreover, these thresholds were comparable between all groups, suggesting that habituation was well established. Immunohistochemistry measurements were not performed for other drugs and for all losmapimod doses tested as the aim of our immunohistochemistry study was to provide a preliminary investigation of the targeted site of action of losmapimod. In line with this, neurones in DRG were only marked with NeuN, a nucleic and cytoplasmic marker,^38^ but not with distal axon cylinder or dendritic ramification. Lastly, female animals were not included in this study. Information regarding the influence of sex hormones on the p38 MAPK pathway is controversial.^39^^,^^40^ Choosing only male animals eliminated the potential effects of sex on our results while making the study more homogeneous. However, this remains a broad topic, and further studies wholly dedicated to it could be very useful.

In conclusion, losmapimod appears to be a promising drug for acute pain control with antinociceptive properties, but further studies are necessary to confirm these results and to test the drug in other pain models. Drug testing in humans is necessary to confirm the clinical efficacy of losmapimod and its apparently low risk of side-effects.

## Authors' contributions

Conception and design of the experiments: MS, LH, MBR, JXM, DB.

Conduct of experiments: MS, LH.

Statistical analysis: MS, JXM.

Writing of the manuscript: LH, MBR.

Drafting and critical revision of the manuscript: MS, JXM, DB.

All authors helped in the interpretation of data and contributed to writing the manuscript. All authors approved the final approval of the version and agree to be accountable for all aspects of the work in ensuring that questions related to the accuracy or integrity of any part of the work are appropriately investigated and resolved.
